# Caseinate–Carboxymethyl Chitosan Composite Edible Coating with Soybean Oil for Extending the Shelf Life of Blueberry Fruit

**DOI:** 10.3390/foods14152598

**Published:** 2025-07-24

**Authors:** Amal M. A. Mohamed, Hosahalli S. Ramaswamy

**Affiliations:** Department of Food Science and Agricultural Chemistry, Macdonald Campus of McGill University, 21111 Lakeshore Road, Ste Anne de Bellevue, QC H9X 3V9, Canada; amal.mohamed@mail.mcgill.ca

**Keywords:** edible coating, postharvest preservation, natural polymers

## Abstract

Utilizing edible films/coatings promises to extend the shelf life of fruits by controlling various physiological parameters (e.g., respiration and transpiration rates), maintaining firmness, and delaying fruit senescence. The influence of composite-based edible coatings made from sodium or calcium caseinate: carboxymethyl chitosan (75:25) on the postharvest quality of fresh blueberries was assessed over a 28-day storage period, on the basis of weight loss and changes in pH, firmness, color, titratable acidity, soluble solids content, mold and yeast count, and respiration rate. The pH of the blueberries increased over the period of storage, with significant differences observed between uncoated and coated (e.g., pH was 3.89, 3.17, and 3.62 at the end of the storage time for uncoated, Ca 75-1% SO, and Na 75-1% SO, respectively. Desirable lower pH values at the end of storage were obtained with the calcium caseinate formulations. Over the duration of storage, other quality parameters (e.g., firmness) were better retained in coated fruits compared to the uncoated (control) one. At the last storage day, the firmness of the uncoated sample was 0.67 N·mm^−1^ while the sodium and calcium caseinate was 0.63 and 0.81 N.mm^−1^, respectively. Moreover, the microbial growth was reduced in coated fruits, indicating the effectiveness of coatings in preserving fruit quality. The mold /yeast count was 1.4 and 2.3 log CFU/g for CaCa 75-1% SO and NaCa 75-1% SO compared with uncoated with 4.2 log CFU/g. Adding soybean oil to the caseinate–carboxymethyl chitosan composite edible coating has the potential to positively influence retention of various quality parameters of blueberries, thereby extending their shelf life and maintaining overall quality. Further research could explore the optimization of coating formulations and application methods to enhance their effectiveness in preserving fruit quality during storage.

## 1. Introduction

From the family Ericaceae, blueberries (*Vaccinium* ssp.) include approximately 450 species [1]. These widely consumed, small, dark blue fruits are of significant economic value and offer several health benefits; accordingly, their popularity has increased over the last decade. Among blueberry varieties, highbush blueberries (*Vaccinium corymbosum* L.) are the most commonly cultivated commercial type. The blueberries can be stored for 10 to 40 days under refrigeration depending on factors such as harvest maturity and storage condition [2]. However, due to water loss and ensuing decomposition by molds, fresh blueberries are highly susceptible to postharvest changes.

Fresh fruits and vegetables are essential components of a healthy diet, like minor proteins, carbohydrates, minerals, fiber, and other nutrients. It is widely agreed that consuming a variety of fruits and vegetables will be beneficial and needed for health and wellbeing [3]. An intake of 300–600 g per day (200–600 g of vegetables and 100–300 g of fruits) has been recommended to meet various combinations of health and environmental objectives [4]. The World Health Organization (WHO) recommends that adults should consume at least 400 g of fruits and vegetables daily [5]. Unfortunately, postharvest losses, due to issues like pathogen and microbial growth, physical damage, respiration, and nutrient loss, can lead to the culling of up to half of fruits and vegetables at about 45–55% during handling and storage. Other foods like cereals, seafood, and meats, however, experience lower losses at approximately 30%, 35%, and 20%, respectively [6].

To reduce such losses and maintain the quality of fruits and vegetables during storage and transportation, studies have tested various safe and convenient preservation techniques to reduce deterioration and extend shelf life, e.g., edible coatings [7], air conditioning [8], irradiation [9], and pressure conditioning [10]. Aligning with the growing demand for sustainable packaging solutions, edible film coatings have been widely implemented as a smart solution for maintaining food products’ freshness. Materials like protein, lipids, polysaccharides, and their combination can create a protective barrier film around the food, preventing moisture loss, oxidation, and microbial contamination [11].

The main challenge in fresh food preservation is the deterioration of quality due to moisture loss, oxidation, and microbial contamination, which ultimately reduces the value of the foods.

Coating techniques have emerged as a promising solution to address this challenge. This technique involves the application of protective coatings to the surface of food, offering a barrier against moisture loss, microbial growth, and other deteriorative factors. The global use of biopolymer materials as edible coatings has markedly gained interest in the field of food preservation and has emerged as a promising tool to extend storage and shelf life of different fruits while maintaining their quality [12,13]. The use of edible coatings has been applied to a wide range of fruit, e.g., figs (*Ficus carica* L.) [14], sweet cherries (*Prunus avium* L.) [15], mushrooms [16], mangoes (*Mangifera indica* L.) [17], fresh-cut apples [18], strawberries, and raspberries (*Rubus idaeus* L.) [19].

In recent years, increasing consumer demand for safe, healthy, and high-quality foods has led to innovative preservation methods. Edible coatings are among these methods, which offer the advantages of easy operation, low-cost, and effectiveness. The application of edible coatings has been widely applied to prolong the shelf life of fruits and vegetables [20]. Carboxymethyl chitosan (CMCH) is a modified carbohydrate derived from chitosan. Due to the abundance of carboxyl groups, CMCH exhibits better solubility in water and biodegradability as compared with chitosan, which has poor solubility in water but high solubility in acidic media [21]. Blending CMCH with other polymers can be used effectively in edible film packaging applications because of its resistance to water and oxygen [22]. However, CMCH films exhibited are brittle, strongly hydrophilic, and have poor mechanical properties, limiting their use in food packaging applications [23]. Alternatively, caseinate-based edible films are water-soluble, highly transparent, and offer good mechanical properties due to its formation of weak intermolecular interactions, thanks to its random coil conformation [24]. In developing composite edible films and coatings, multiple biopolymers have been combined. Blending edible films is needed due to its multifaceted benefits. Blended films play a crucial role in food preservation by providing barriers against moisture and oxygen.

Lipids are fatty acid polymers consisting of long chains of nonpolar hydrocarbons. Furthermore, lipid coatings provide an excellent barrier against oxygen and moisture, allowing food to be kept fresh for longer periods of time. These edible lipid films and coatings also offer nutritional and functional qualities. However, previous studies have largely focused on different oil types (e.g., sunflower, coconut, olive, as well as linseed and rosehip oil) [25,26]. Soybean oil provides a cost-effective and widely available lipid phase that can enhance barrier properties and bioactivity through its unsaturated fatty acid profile. While chitosan–caseinate coatings have been explored, no investigation exits on the incorporation of soybean into this combination matrix for blueberry preservation.

This is the first attempt to incorporate soybean oil in blending caseinate–carboxymethyl chitosan in order to enhance the physicochemical properties of blueberry coatings. Recent studies (Volpe [27] as well as Mohamed and Ramaswamy [28]) found that combining chitosan and sodium caseinate enhances the mechanical properties of films compared with control films and the addition of oil improves barrier properties. Based on these findings, the present study aims to develop a novel composite edible coating.

While edible coatings are not designed to completely replace traditional packaging methods, they can enhance food protection when used in combination with conventional approaches. The main objective of the present study was to use the composite edible coating concept to extend the shelf life of blueberries while preserving their physicochemical characteristics. This study is aimed at improving the postharvest stability of blueberries by utilizing the composite coating that reduces water loss and microbial spoilage, thereby prolonging shelf life and reducing food waste. This was to be achieved by using a functionally composite film made from a combination of caseinates–carboxymethyl chitosan, incorporated with soybean oil.

## 2. Materials and Methods

### 2.1. Materials

Blueberry fruits were purchased from a local market. Carboxymethyl chitosan powder was obtained from Nutrakey Industries, Inc. (Qingdao, China), while sodium caseinate (abbreviation, NaCa, 92.0% protein) and calcium caseinate (abreviation, CaCa, 92.6% protein) were obtained from CALDIC Canada Inc. (Mississauga, ON, Canada). Glycerol and soybean oil were obtained from Bulk Apothecary (Aurora, OH, USA). Other chemicals were purchased from Sigma-Aldrich (Oakville, ON, Canada), including peptone water, plate count agar, phenolphthalein, potato dextrose agar, sodium hydroxide, sodium hydrochloride, and Tween 20.

### 2.2. Preparation of Coating Solutions

**Figure 1:**
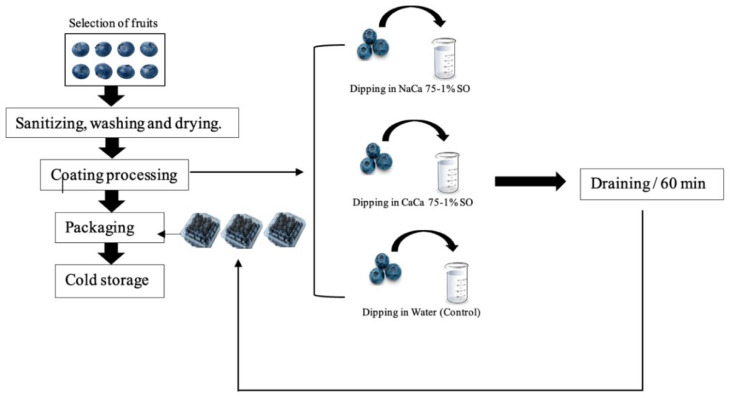
Process flow diagram of fresh blueberry fruit coating with a caseinates–carboxymethyl chitosan (CAs-CMCH) composite-based edible film with 1% soybean oil.

The edible coating was prepared in two different forms: (i) sodium caseinates–carboxymethyl chitosan (NaCa-CMCH) with 1% of soybean oil and (selected based on the reported ability to improve emulsion stability and barrier properties) [28] (ii) calcium caseinates–carboxymethyl chitosan (CaCa-CMCH) with 1% of soybean oil. The ratio of caseinates to carboxymethyl chitosan was 75:25 *w*/*w* or both sodium and calcium caseinate forms, which is based on the previous optimization study and supported by other findings. At this ratio, the film showed good integrity as well as mechanical and water vapor-permeable properties. Both (Na and Ca) caseinates solutions contained 8 g CA (100 mL^−1^) and 3 g CMCH (100 mL^−1^) of distilled water. This concentration was selected based on the optimization and evaluation of the physicochemical and functional characteristics of edible films before their application as edible coatings. To generate the coating solution, 75 mL of caseinates solution and 25 mL of carboxymethyl chitosan were mixed for 15 min using a homogenizer (Biospec Products, Bartlesville, OK, USA, INS. Model 985-370) at room temperature. Glycerol 0.5% *w*/*w* and Tween 20 (0.1% *w*/*w*) were added to both solutions, and these were mixed for 10 min. Soybean oil at 1% (SO) was then homogenized into all of the solutions before centrifugation at 13,000 rpm for 15 min. The coating solutions were termed NaCa 75-1% SO and CaCa 75-1% SO for sodium and calcium caseinates–carboxymethyl chitosan mixtures bearing 1% soybean oil. For the control samples, distilled water was used (uncoated) as the dipping solution (procedure illustrated in Figure 1).

### 2.3. Sample Preparation

Blueberry fruits (≈3 kg) were selected for study based on uniformity of size and color without damage or fungal infection, then sanitized with sodium hypochlorite water solution (0.1 g kg^−1^), rinsed with distilled water, and dried on absorbing papers. The fruits were divided into three groups: (i) those dipped in NaCa 75-1% SO, (ii) those dipped in CaCa 75-1% SO, and (iii) the control sample (non-coated) was dipped in distilled water. The dipping process took about 5 min, followed by drying of samples on a ventilated surface for 60 min. The coated and uncoated blueberry samples were placed in clear polyethylene terephthalate trays and stored at 4 °C for 28 days. The shelf life study was carried out over a 28-day storage period because it reflects the typical shelf life of blueberries under refrigeration. Quality tests were performed every four days to observe gradual changes while keeping the number of tests manageable. For microbiological analysis, we selected days 0, 4, 10, 20, and 28 to capture key stages of microbial growth while limiting the number of samples used, based on practical and standard lab guidelines.

### 2.4. Shelf Life Studies

All experiments were at least carried out in triplicate. The following analyses were carried out to examine the quality changes in the samples every four days for 28 days. In this study, the addition of soybean oil (1%) was effective in improving the edible film and coating qualities of the caseinate–CMCH coating, a critical innovative aspect of this research [28]. Soybean oil was specifically selected because of its high concentration of unsaturated fatty acids, which enhances the hydrophobicity and moisture barrier property of the coating. This composition significantly inhibits weight loss and maintains fruit firmness in storage. To our knowledge, the combination of these polymers with soybean oil in the form of a composite blueberry coating is a new technique, which has not yet been reported. This new composition has promising potential for postharvest preservation of fresh fruit.

### 2.5. pH, Brix, Titratable Acidity, and Weight Loss

The pH of a blueberry fruit was measured at room temperature using a digital pH meter (Brinkman Co., Mississauga, Ontario, Canada). Approximately 50 g of the samples were blended with 150 mL distilled water [29]. Total soluble solids (TSS) concentration in the samples was determined by a digital refractometer (ATAGO, Model. PAL-α, Pro-lab scientific, 1974, Laval, QC, CA) and expressed on the Brix scale 0–85%.

Titratable acidity (TA) was measured following the AOAC standard 942.15 method [30]. Values were expressed as grams of citric acid per 100 mL of sample. Weight loss (*WL*) was measured as the percentage decrease from the original fresh weight. Samples were weighed at time zero to day 28 using a digital analytical balance as described by [31]. The percentage weight loss was calculated as follows:

(1) $$WL\;(\%)=100\times \frac{{FW}_{t=0}-{FW}_{t=x}}{{FW}_{t=0}}$$

where ${FW}_{t=0}$ is the initial fresh weight of the blueberry sample (*t* = 0), and ${FW}_{t=x}$ is the fresh weight of the blueberry sample at sampling day *x.*

### 2.6. Textural Analysis

Measuring food texture before and after treatment is a critical step in food processing and quality control. It involves assessing how the texture of a food product has been modified due to various treatments. Texture was measured to determine the hardness (i.e., maximum force during the first compression) of 4 °C-stored uncoated and coated samples. The instrument used was a TA XT Plus Texture Analyzer (Texture Technologies Corp., Scarsdale, NY, USA) fitted with 25 mm diameter cylindrical probe and 50 kg load cell in compression mode. The adopted test procedure of Márquez et al. [32] was pre-test and post-test speed of 2.0 mm s^−1^, test speed of 1.0 mm s^−1^, and deformation to original height of 75%. Crosshead speed for both compressions (“bites”) was kept at 5 mm s^−1^. All treatments were tested in at least 15 individual samples, the values of which were read in terms of hardness.

### 2.7. Color Measurements

Color attributes L* (lightness), a* (green–red axis), and b* (blue–yellow axis) were determined at room temperature on a calibrated Minolta Chroma Meter (Minolta Corp., Ramsey, NJ, USA). Ten samples of blueberry per treatment (coated and uncoated samples) were recorded at room temperature.

### 2.8. Respiration Rate (RR)

Respiration was measured every four days during 28-day storage on about 50 g blueberries in a sealed Plexiglas chamber (18 × 12 × 27 cm) at room temperature. CO_2_ concentration was monitored continuously for two hours using a CO_2_ sensor (ACR Systems Inc., St-Laurent, QC, Canada) connected to a Smart Reader Plus 7 data logger. The respiration rate was obtained from the regression slope of gas concentration versus time and the respiration rates of the blueberries were determined as mL CO_2_ kg^−1^·h^−1^ [33].

### 2.9. Microbial Growth

Microbiological enumeration was carried out on uncoated and coated blueberry fruit samples on days 0, 4, 10, 20, and 28. Approximately 10 g of each sample were homogenized with 90 mL of sterile peptone water (0.1% *w*/*w*). Serial dilutions (10^−1^ to 10^−4^) were plated on selective media. PCA (plate count agar) was used for aerobic mesophilic bacteria counts followed by incubation at 30 °C for 48 hrs. Yeast and mold counts were determined by the surface plate method using PDA (potato dextrose agar), with incubation at 25 °C for 2 days. All tests were performed in triplicate.

### 2.10. Data Analyses

SPSS V31 software (SPSS, 2023) was used in statistical analyses to determine the effect of the coating and storage time on quality and shelf life parameters of fresh blueberries. Differences between treatments were tested for significance by one-way ANOVA. Significantly different means (*p* ≤ 0.05) were separated by the Duncan test.

## 3. Results and Discussion

### 3.1. pH, Soluble Solids Content, Titratable Acidity, and Weight Loss

**Figure 2:**
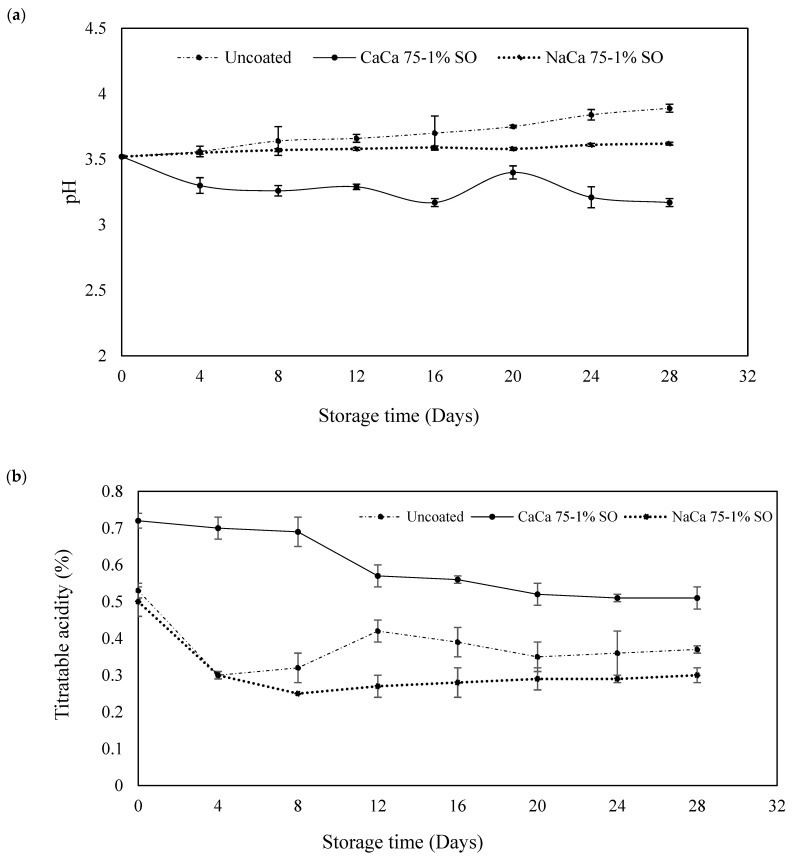

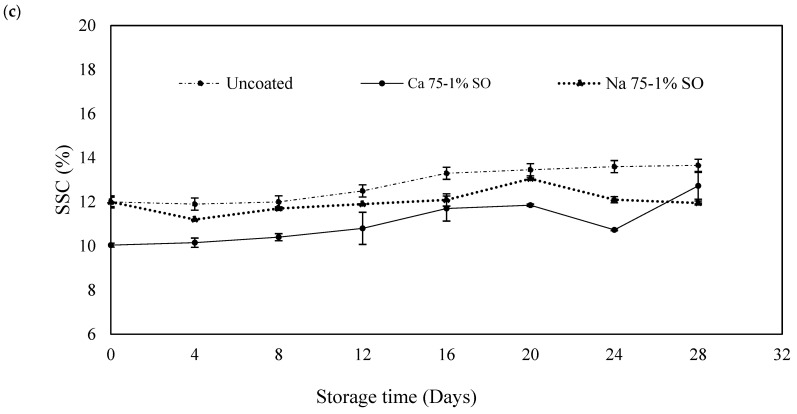
pH (**a**), titratable acidity (**b**), and soluble solids content (SSC) (**c**) of uncoated and coated blueberries with caseinates–carboxymethyl chitosan (CAs-CMCH) composite-based edible coatings with 1% soybean oil (SO).

The pH, SSC, and TA were examined to assess the metabolic status of blueberries, a key factor in fruit quality. The pH increased during storage for both uncoated and coated samples, although NaCa 75-1% SO had numerically higher pH values than uncoated treatments, but the differences were not statistically significant (*p* > 0.05) until day 20 (Figure 2a). Compared with CaCa 75-1% SO results, which showed a lower pH value, the ranges of pH for uncoated and NaCa 75-1%SO-coated fruits were 3.52–3.89 and 3.52–3.62, respectively, while the pH reading for CaCa 75-1% SO ranged between 3.52 and 3.17 during the storage period.

The CaCa 75-1% SO-coated sample showed the highest TA values compared to the other samples (Figure 2b). Overall, TA values declined with storage time, which could be the result of the use of organic acids such as citric acid in the respiration process. Organic acids are commonly metabolized via the tricarboxylic acid (TCA) cycle to produce energy. This respiratory utilization often results in a decline in titratable acidity over time during storage (Fan et al., 2022) [34]. The NaCa 75-1%SO showed lower TA values; therefore, this coating could apparently help retain TA in blueberries.

The TSS of all treatments increased considerably until 16 days of storage (Figure 2c). The increase was significantly (*p* ≤ 0.05) greater in the control samples than in coated samples. The lower TSS in the treated fruits could be the result of the different effects of the coated materials. On day 28 of storage, the control fruit showed higher TSS values as compared with the coated samples, and a significant difference was observed between the coated samples. A similar trend was observed by Abugoch et al. [35], who used chitosan incorporated with quinoa protein and sunflower oil to improve the shelf life of blueberries. They found that the uncoated (control) fruit showed a higher pH value than the coated samples. The higher TSS values in the control fruit after 21 days of storage could be attributed to fast acid metabolism converting starch and acid to sugar, resulting in a higher pH and TSS [35].

**Figure 3:**
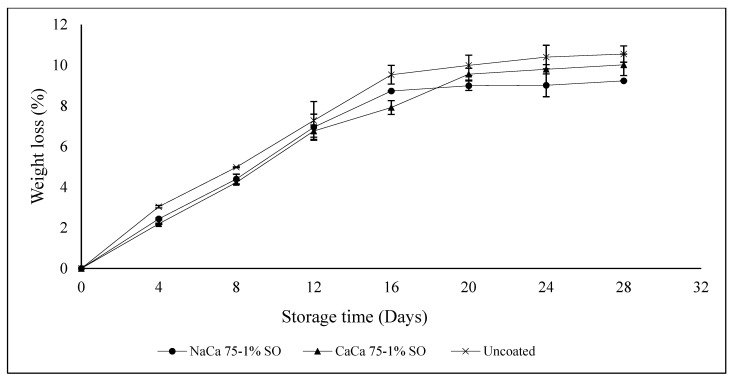
Blueberries weight loss (%) during storage time for uncoated and coated samples with caseinates–carboxymethyl chitosan (CAs-CMCH) composite-based edible with 1% soybean oil (SO).

Weight loss during storage is a critical factor in determining blueberry quality, with 5% to 8% weight loss being the level at which blueberries can become unmarketable [36]. Fruits and vegetables’ weight loss during storage is generally due to migration of the water from the food to the surrounding atmosphere. Moisture loss is also caused by the gradient of water vapor pressure that occurs between different locations in the cell [37]. However, variations in temperature and relative humidity may influence the vapor pressure difference between blueberries and the surrounding environment, resulting in non-significant weight losses. All samples demonstrated some weight loss during the first 16 days of storage, with the uncoated sample (control) showing the highest percentages followed by NaCa 75-1% SO and CaCa 75-1% SO, with 9.53%, 8.73%, and 7.92%, respectively (Figure 3). This demonstrated the effectiveness of edible coating on blueberrry fruits in reducing moisture loss, with uncoated samples having the highest loss in weight, at 10.55% after 28 days of storage. The coated sample with CaCa 75-1% SO better maintained its weight during storage.

### 3.2. Textural Analysis

**Figure 4:**
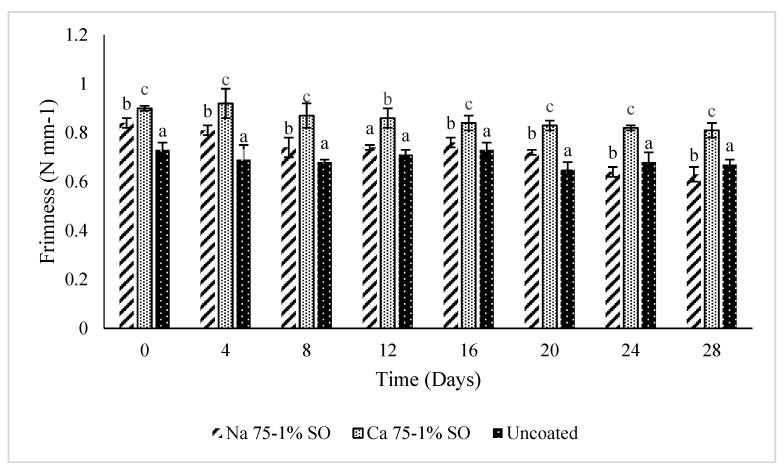
Firmness (N) of uncoated and coated blueberry samples with caseinates–carboxymethyl chitosan (CAs-CMCH) composite-based edible with 1 %soybean oil (SO). Data not sharing the same letter are significantly different (*p* < 0.05).

Texture is a key indicator of the freshness and ripeness of fruits and vegetables, as well as consumer acceptance, as it is related to metabolic and water content changes. Blueberries are non-climacteric fruits, meaning that they do not continue ripening after harvest and have limited respiration-related softening. However, the degradation in the texture is mostly due to moisture loss and enzymatic activities [38]. As expected, the texture of the samples softened with storage time (Figure 4). Storage time and treatment had effects on the firmness of all the samples. Coated samples showed better firmness than uncoated samples, which showed significant softening. Up to 20 days of storage, the blueberry samples for both coated and uncoated treatments showed a significant change in firmness (*p* ≤ 0.05). However, the CaCa 75-1% SO coating offered better firmness than either the NaCa 75-1% SO coating or the uncoated fruit. After day zero, all samples stared with relatively high firmness, with CaCa 75-1% SO showing the highest value at around 0.9 Nmm^−1^. By last day of storage, CaCa 75-1% SO still retained a firmness of 0.8 Nmm^−1^, while NaCa 75-1% SO dropped to 0.63 Nmm^−1^ and the uncoated to 0.67 Nmm^−1^. This was attributable to the coating materials leading to good maintenance of the outer cellular membrane of the fruits. These findings corroborated the transpiration results. Firmness is a crucial quality of fruits in terms of their appeal to consumers and market value. Blueberries usually become softer after harvesting, which lowers their shelf life. Using a coating process can positively affect fruit texture, leading to an extension of their shelf life. Many studies have investigated the effect of applying an edible coating to fruits. Marquez-Villacorta et al. [39] reported that, compared with an uncoated control, the texture of coated blueberries was improved by using an edible coating bearing an essential oil. Similar findings were mentioned by others [40,41].

### 3.3. Color

**Figure 5:**
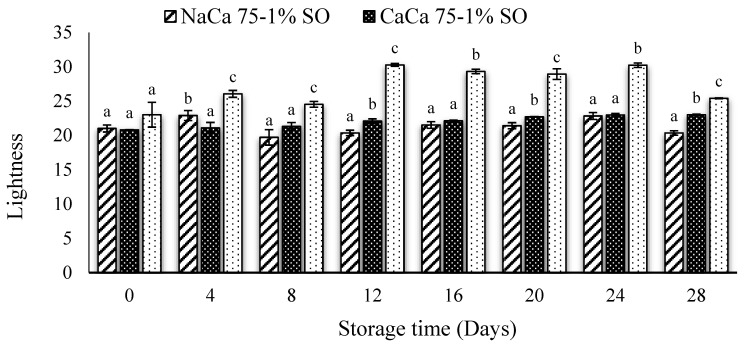
Lightness of uncoated and coated blueberry samples with caseinates–carboxymethyl chitosan (CAs-CMCH) composite-based edible with 1% soybean oil (SO). Data not sharing the same letters are significantly different from each other (*p* < 0.05).

**Figure 6:**
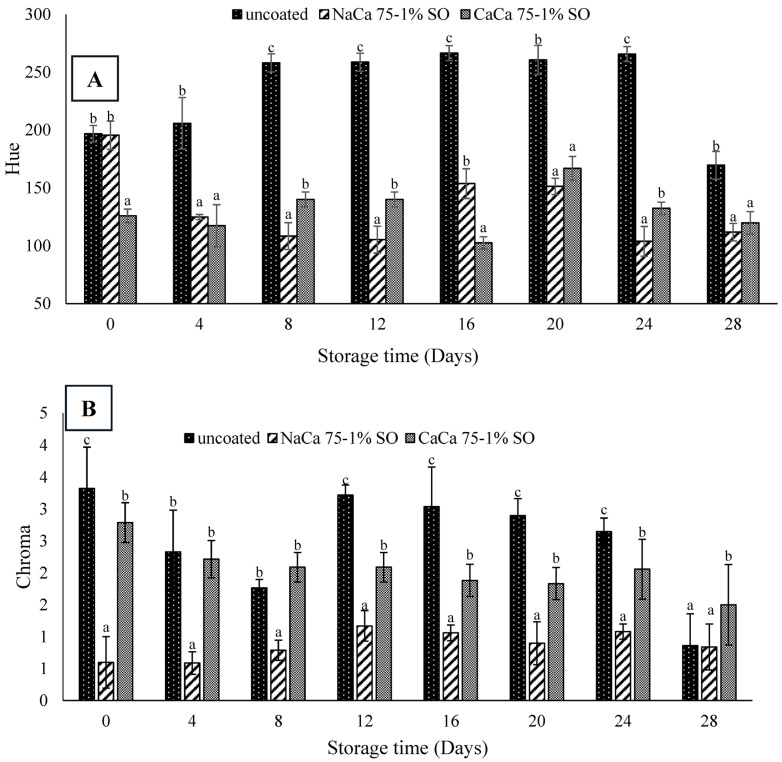
Hue (**A**) and chroma (**B**) of uncoated and coated blueberry samples with caseinates–carboxymethyl chitosan (CAs-CMCH) composite-based edible with 1% soybean oil (SO). Data not sharing the same letters are significantly different from each other (*p* < 0.05).

The color transformation of blueberries during the storage is a significant postharvest quality parameter. Table 1 and Figure 5 and Figure 6 show these color changes by measuring lightness (L*), yellowness (a*), redness (b*), hue, and chroma of uncoated and coated samples over 28 days of the storage period. The lightness (L*) of the coated samples showed only a slight change over time, whereas uncoated samples exhibited an increase in lightness from day 12 to 24, then a decrease at 28 days. In general, coated samples led to less lightness compared to uncoated samples (*p* ≤ 0.05), which could be attributed to the glossy effect of the coated surface. Abugoch et al. [35] reported that the control samples showed greater lightness as compared with samples treated with an edible coating made of chitosan/quinoa protein, with sunflower oil incorporated in extending the shelf life of blueberries.

From Table 1, the coating process significantly affected (*p* ≤ 0.05) the degree of redness (a*) and yellowness–blueness (b*), while similar results were found for the uncoated samples (Table 1). The initial (a*) value of the uncoated fruit at day 0 was 0.47 and it decreased up to 0.10 by the last day of storage. Comparatively, the CaCa 75-1% SO coating offered the highest value of (a*) at the end of storage period (1.10). The b* value exhibited a similar trend to the (a*) results, with uncoated samples having higher values at the beginning of storage and a lower value at the end of the storage period. The intensity of blue color was more intense in the uncoated samples than the coated ones, as indicated by higher hue values (Figure 6). The chroma results followed the same trend as hue, being higher in the uncoated sample than in the coated one. However, no significant differences (*p* ≥ 0.05) in the hue value was found between the NaCa 75-1% SO and CaCa 75-1% SO treatments from day 4 until day 28, while the chroma results showed significant differences (*p* ≤ 0.05), with CaCa 75-1% SO exhibiting higher values. In comparison to the present research findings, several studies have explored the impact of coatings on the color parameter of blueberries. Tahir et al., [42] found that using gum Arabic with African baobab pulp extract for coating blueberries had an effect on color parameters. L* decreased with storage time for both control and coated samples over 21 days, which could be attributed to changes in anthocyanin content and the loss of surface moisture. Anthocyanins are responsible for the blue dark color and due to enzymatic activity, pH changes, and oxidation, which will lead to degradation pf the dark skin and a lower L value. In another study, Mannozzi et al. [43] reported that, in general, uncoated (control) samples exhibited higher L* (lightness) than samples treated with sodium alginate and pectin as an edible coating.

### 3.4. Respiration Rate

**Figure 7:**
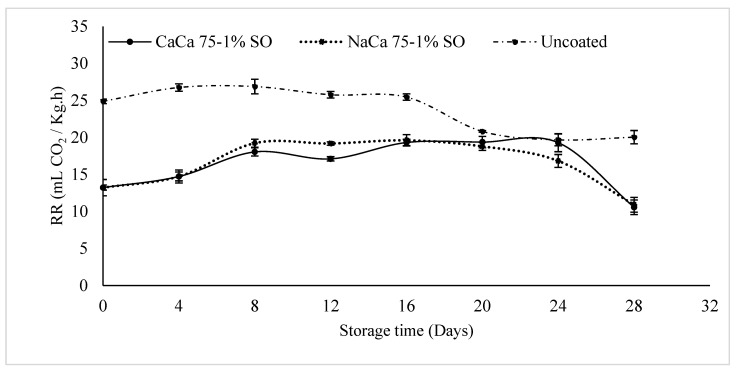
Respiration rate (RR) of uncoated and coated blueberry samples with caseinates–carboxymethyl chitosan (CAs-CMCH) composite-based edible with 1% soybean oil (SO).

A fundamental physiological process in the cell, respiration rate refers to the exchange of gases between an organism and its surrounding environment. The respiration rate of coated and uncoated blueberry samples is shown in Figure 7. According to the relationship between the respiration rate and shelf life of fruits and vegetables, higher respiration rates lead to a shorter storage time. Until day 16 of storage, the respiration rate of uncoated samples (control) was higher than that of coated samples. The former then decreased but continued to be higher than that of the coated blueberries. The respiration rate of the CaCa 75-1% SO and NaCa 75-1% SO edible coating treatments did not show any significant differences (*p* > 0.05), both increasing until day 24, then declining at day 28 (the end of the storage time). Although the coated samples showed lower respiration rates, in all samples, the release of CO_2_ showed no significant changes (*p* > 0.05) from day 4 until day 24. However, this could be due to diminished metabolic activity or to the coating reducing CO_2_ permeability. With respect to the respiration rate during storage, there was a gradual increase at the beginning and this increase is due to high postharvest activity and possible stress from handling and coating. From day 8 to 24 of storage, the RR stabilized as the fruit becomes adjusted to the storage condition. Then, after day 24, their respiration starts to drop, likely because of aging, decreased enzyme activity, or lack of respiratory materials.

### 3.5. Microbial Growth

Microbial spoilage has been a significant concern for the food industry as it can affect the quality and shelf life of food, as well as pose potential health risks to consumers. Postharvest shelf life losses of blueberry fruits due to microbial spoilage was determined. In uncoated samples, yeast and mold were detected starting from day 10 (Table 2), the count thereafter increasing till the end of the storage time. Values consistently ranged between 2.2 and 4.2 log 10 cfu g^−1^. The coated samples (CaCa 75-1% SO and NaCa 75-1% SO) both remained under the detection threshold until day 20. In terms of the growth of mesophilic aerobic bacteria, significant differences were found between the samples for each treatment time. Uncoated samples showed detectable levels of bacteria after 4 days. In general, the coating process reduced yeast, mold, and mesophilic bacteria counts compared with the control (uncoated) fruit. Uncoated samples exhibited a higher yeast and mold load compared to the coated ones. The CaCa 75-1% SO coating led to lower microbial counts than the NaCa 75-1% SO coating. However, in general, the antimicrobial coating’s properties can delay or stop microorganisms from growing on food surfaces by creating a physical barrier and releasing bioactive compounds. These led to limiting their ability to access oxygen and nutrients, or they actively disrupt microbial cells [44]. These helps extend shelf life, improve food safety, and reduce spoilage. Moreover, antimicrobial activity of the coating is attributed to the synergy between CMCH and soybean oil. CMCH is active against microorganisms via electrostatic interaction with microbial membranes, inducing greater permeability and cell death [45]. However, the incorporation of soybean oil increases hydrophobicity of the coating and may rupture microbial membranes through lipid–lipid interaction directly, which represses microbial viability and increases barrier property [46].

## 4. Conclusions

This study highlights the effectiveness of a composite edible coating of caseinates–carboxymethyl chitosan, incorporated with soybean oil, in maintaining the postharvest quality and shelf life of blueberry fruits. Applying edible coatings to fresh blueberry fruit can create a protective layer on the fruit, extending shelf life by preventing gas and water transfer. By adding lipids to a composite edible coating, one can enhance the coating’s moisture barrier properties. This technology shows the potential for reducing postharvest losses and improving the preservation of food quality and safety, with a focus on extending the shelf life of food. Coating of fruit showed a positive impact on various quality parameters, mainly texture and microbial growth. Overall, the findings suggest that the use of composite-based edible coatings with soybean oil can positively influence various quality parameters of blueberries, including pH, TA, weight loss, texture, color, respiration rate, and microbial growth, thereby extending their shelf life and maintaining overall quality. Further research could explore optimization of coating formulations and application methods to enhance their effectiveness in preserving fruit quality during storage.

## Figures and Tables

**Table 1:** The a* and b* value of uncoated and coated blueberry samples with caseinates–carboxymethyl chitosan (CAs-CMCH) composite-based edible with 1%soybean oil (SO).

a*								
	Day 0	Day 4	Day 8	Day 12	Day 16	Day 20	Day 24	Day 28
Uncoated	0.47 ± 1.19 ^a^	0.19 ± 0.18 ^b^	0.28 ± 0.08 ^c^	0.40 ± 0.11 ^d^	0.18 ± 0.17 ^b^	0.45 ± 0.12 ^a^	0.18 ± 0.16 ^b^	0.10 ± 0.29 ^e^
NaCa75−1%SO	0.20 ± 0.24 ^a^	0.27 ± 0.11 ^b^	0.23 ± 0.09 ^a^	0.28 ± 0.14 ^b^	0.15 ± 0.21 ^c^	0.55 ± 0.40 ^d^	0.25 ± 0.04 ^e^	0.32 ± 0.16 ^f^
CaCa75−1%SO	0.53 ± 0.50 ^a^	0.73 ± 0.30 ^b^	0.73 ± 0.37 ^b^	0.79 ± 0.20 ^c^	1.24 ± 1.03 ^d^	1.40 ± 0.34 ^e^	1.23 ± 0.60 ^d^	1.10 ± 0.34 ^f^
b*								
	Day 0	Day 4	Day 8	Day 12	Day 16	Day 20	Day 24	Day 28
Uncoated	−3.08 ± 0.99 ^a^	−2.31 ± 0.64 ^b^	−1.74 ± 1.13 ^c^	−3.17 ± 1.89 ^d^	−3.03 ± 0.61 ^a^	−2.85 ± 0.96 ^a^	−2.63 ± 0.22 ^e^	−0.61 ± 0.81 ^f^
NaCa75−1%SO	−0.45 ± 0.52 ^a^	−0.47 ± 0.28 ^a^	−0.74 ± 0.18 ^b^	−1.12 ± 0.28 ^c^	−1.04 ± 0.14 ^d^	−0.14 ± 0.81 ^e^	−1.05 ± 0.12 ^d^	−0.77 ± 0.22 ^f^
CaCa75−1%SO	−1.66 ± 0.62 ^a^	−1.98 ± 1.12 ^b^	−1.72 ± 1.36 ^c^	−1.72 ± 1.30 ^c^	−0.51 ± 1.55 ^d^	−1.52 ± 0.98 ^e^	−1.35 ± 0.82 ^f^	−1.33 ± 0.44 ^f^

Means (±) standard deviation values (*n* = 3) followed by a different letter within the same row are significantly different (*p* ≤ 0.05) by DSL test.

**Table 2:** Microbial growth of uncoated and coated blueberries samples with caseinates–carboxymethyl chitosan (CAs-CMCH) composite-based edible with 1% soybean oil SO.

Yeast and Mold Counts (log 10 cfu g^−1^)					
	**Day 0**	**Day 4**	**Day 10**	**Day 20**	**Day 28**
Uncoated	nd	2.2 ± 0.2 ^a^	3.1 ± 0.4 ^a^	3.8 ± 0.2 ^a^	4.2 ± 0.3 ^a^
NaCa 75-1% SO	nd	0	0	1.3 ± 0.1 ^b^	2.3 ± 0.4 ^b^
CaCa 75-1% SO	nd	0	0	0.9 ± 0.5 ^c^	1.4 ± 0.2 ^c^
Mesophilic aerobic bacteria count (log 10 cfu g^−1^)					
	**Day 0**	**Day 4**	**Day 10**	**Day 20**	**Day 28**
Uncoated	nd	2.9 ± 0.1 ^a^	3.3 ± 0.2 ^a^	4.1 ± 0.1 ^a^	4.2 ± 0.3 ^a^
NaCa 75-1% SO	nd	1.2 ± 0.2 ^b^	1.5 ± 0.4 ^b^	2.1 ± 0.2 ^b^	2.3 ± 0.3 ^b^
CaCa 75-1% SO	nd	0	0	1.1 ± 0.3 ^c^	1.4 ± 0.1 ^c^

^a–c^ Different letters in the same column are significantly different samples at a 95% confidence level and obtained from three replicates for each treatment time. Counts are expressed in log10 cfu g^−1^ (± standard deviations).

## Data Availability

The original contributions presented in this study are included in the article. Further inquiries can be directed to the corresponding author.
